# Supplementary material for: Nuclear division phenotypes in Sporidiobolales and Trichosporonales
Source: Microbiol Spectr. 2025 Oct 7;13(11):e01327-25. doi: 10.1128/spectrum.01327-25 (PMC12584667; doi:10.1128/spectrum.01327-25)
Supplement: Supplemental material — Fig. S1; Tables S1 and S2. [file spectrum.01327-25-s0001.pdf]

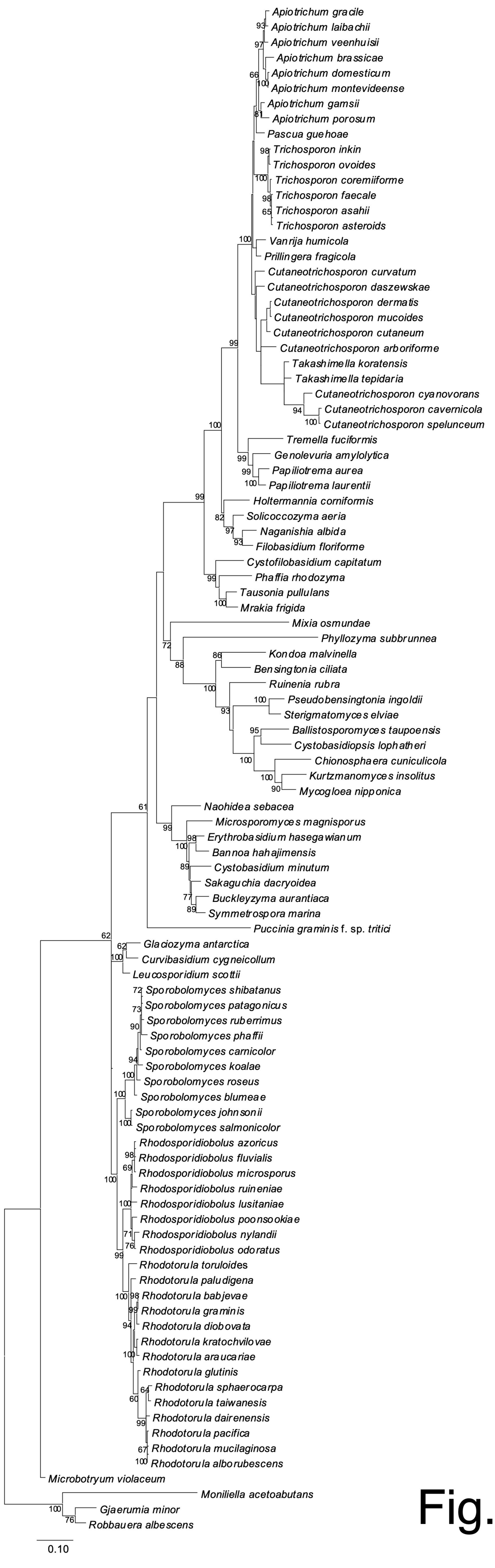
Apiotrichum gracile
Apiotrichum laibachii
Apiotrichum veenhuisii
Apiotrichum brassicae
Apiotrichum domesticum
Apiotrichum montevideense
Apiotrichum gamsii
Apiotrichum porosum
Pascua guehoae
Trichosporon inkin
Trichosporon ovoides
Trichosporon coremiiforme
Trichosporon faecale
Trichosporon asahii
Trichosporon asteroids
Vanrija humicola
Prillingera fragicola
Cutaneotrichosporon curvatum
Cutaneotrichosporon daszewskae
Cutaneotrichosporon dermatis
Cutaneotrichosporon mucoides
Cutaneotrichosporon cutaneum
Cutaneotrichosporon arboriforme
Takashimella koratensis
Takashimella tepidaria
Cutaneotrichosporon cyanovorans
Cutaneotrichosporon cavernicola
Cutaneotrichosporon spelunceum
Tremella fuciformis
Genolevuria amylolytica
Papiliotrema aurea
Papiliotrema laurentii
Holtermannia corniformis
Solicoccozyma aeria
Naganishia albida
Filobasidium floriforme
Cystofilobasidium capitatum
Phaffia rhodozyma
Tausonia pullulans
Mrakia frigida
Mixia osmundae
Phyllozyma subbrunnea
Kondoa malvinella
Bensingtonia ciliata
Ruinenia rubra
Pseudobensingtonia ingoldii
Sterigmatomyces elviae
Ballistosporomyces taupoensis
Cystobasidiopsis lophatheri
Chionosphaera cuniculicola
Kurtzmanomyces insolitus
Mycogloea nipponica
Naohidea sebacea
Microsporomyces magnisporus
Erythrobasidium hasegawianum
Bannoa hahajimensis
Cystobasidium minutum
Sakaguchia dacryoidea
Buckleyzyma aurantiaca
Symmetrospora marina
Puccinia graminis f. sp. tritici
Glaciozyma antarctica
Curvibasidium cygneicollum
Leucosporidium scottii
Sporobolomyces shibatanus
Sporobolomyces patagonicus
Sporobolomyces ruberrimus
Sporobolomyces phaffii
Sporobolomyces carnicolor
Sporobolomyces koalae
Sporobolomyces roseus
Sporobolomyces blumeae
Sporobolomyces johnsonii
Sporobolomyces salmonicolor
Rhodosporidiobolus azoricus
Rhodosporidiobolus fluvialis
Rhodosporidiobolus microsporus
Rhodosporidiobolus ruineniae
Rhodosporidiobolus lusitaniae
Rhodosporidiobolus poonsookiae
Rhodosporidiobolus nylandii
Rhodosporidiobolus odoratus
Rhodotorula toruloides
Rhodotorula paludigena
Rhodotorula babjevae
Rhodotorula graminis
Rhodotorula diobovata
Rhodotorula kratochvilovae
Rhodotorula araucariae
Rhodotorula glutinis
Rhodotorula sphaerocarpa
Rhodotorula taiwanesis
Rhodotorula dairenensis
Rhodotorula pacifica
Rhodotorula mucilaginosa
Rhodotorula alborubescens
Microbotryum violaceum
Moniliella acetoabutans
Gjaerumia minor
Robbauera albescens
0.10

Fig. S1

Fig. S1 Phylogenetic position of strains used in the study

The evolutionary history was inferred by using the Maximum Likelihood method based on the Tamura-Nei model [1]. A discrete Gamma distribution was used to model evolutionary rate differences among sites (5 categories (+G, parameter = 0.4656)). The tree is drawn to scale, with branch lengths measured in the number of substitutions per site. All positions with less than 90% site coverage were eliminated. Bootstrap value less than 60% was not shown.

Three ribosomal RNA gene regions, small subunit (SSU), D1/D2 domains of large subunit (LSU) and internal transcribed spacer regions (ITS, including 5.8S rDNA), were obtained from GenBank database (Table S2). Sequences were aligned with the MAFFT program (https://mafft.cbrc.jp/alignment/server/) using the default parameters and then concatenated. A total of 2517 positions were used to infer the evolutionary history by using the Maximum Likelihood method based on the Tamura-Nei model [1] using MEGA7 [2]. A discrete Gamma distribution was used to model evolutionary rate differences among sites (5 categories (+G, parameter = 0.4656)). All positions with less than 90% site coverage were eliminated.

1. Tamura K. and Nei M. (1993). Estimation of the number of nucleotide substitutions in the control region of mitochondrial DNA in humans and chimpanzees. Molecular Biology and Evolution 10:512-526.

2. Kumar S., Stecher G., and Tamura K. (2016). MEGA7: Molecular Evolutionary Genetics Analysis version 7.0 for bigger datasets. Molecular Biology and Evolution 33:1870-1874.

Supplemental Table 1

Measured values in Fig. 2A, 3A, and 5A are shown.

Fig. 2A

Nuclear division phenotypes are shown in budded cells of 51 Sporidiobolales yeasts. *S. cerevisiae* is an outgroup strain in the analysis.

Sporidiobolales

				All cells				Budded cells			
No.	Species	Strains	Total cell number	Yeast form (%)	Hyphal form (%)	Fission form (%)	Budded cell number	A nucleus in mother cell only (%)	A nucleus in daughter cell only (%)	A nucleus at bud neck (%)	Nuclei in both cells (%)
Asc.	*Saccharomyces cerevisiae*	BY 1438	897	100	0	0	230	74.4	0	5.2	20.4
1	*Rhodosporidiobolus azoricus*	JCM 11251	225	100	0	0	123	65	4.9	5.7	24.4
2	*Rhodosporidiobolus fluvialis*	JCM 10311	299	100	0	0	103	49.5	5.8	10.7	34
3	*Rhodosporidiobolus lusitaniae*	JCM 8547	147	100	0	0	93	60.1	9.7	15.1	15.1
4	*Rhodosporidiobolus microsporus*	JCM 6882	473	100	0	0	239	66.2	4.6	5.4	23.8
5	*Rhodosporidiobolus nylandii*	JCM 10213	177	100	0	0	81	65.4	3.7	7.4	23.5
6	*Rhodosporidiobolus oboratus*	JCM 11641	246	100	0	0	35	46.6	2	0	51.4
7	*Rhodosporidiobolus poonsookiae*	JCM 10207	252	100	0	0	100	77	6	4	13
8	*Rhodosporidiobolus ruineniae*	JCM 1839	296	100	0	0	187	70.6	5.9	1.6	21.9
9	*Rhodosporidiobolus ruineniae*	JCM 8097	358	100	0	0	137	64.2	6.6	5.8	23.4
10	*Rhodotorula alborubescens*	JCM 5352	293	100	0	0	168	60.7	4.2	3	32.1
11	*Rhodotorula araucariae*	JCM 3770	300	100	0	0	175	56.6	9.1	4.6	29.7
12	*Rhodotorula babjevae*	JCM 9279	223	100	0	0	181	77.9	2.8	3.3	16
13	*Rhodotorula dairenensis*	JCM 3774	200	100	0	0	160	59.9	10	13.8	16.3
14	*Rhodotorula diobovata*	JCM 3786	244	100	0	0	161	49.1	9.9	1.2	39.8
15	*Rhodotorula diobovata*	JCM 3787	262	100	0	0	177	53.7	12.4	5.1	28.8
16	*Rhodotorula glutinis*	JCM 8208	146	100	0	0	107	73.8	3.7	1.9	20.6
17	*Rhodotorula graminis*	JCM 3775	284	100	0	0	151	65.5	11.3	1.3	21.9
18	*Rhodotorula kratochvilovae*	JCM 8171	249	100	0	0	145	73.9	5.5	3.4	17.2
19	*Rhodotorula kratochvilovae*	JCM 8172	268	100	0	0	145	66.9	6.2	4.8	22.1
20	*Rhodotorula mucilaginosa*	JCM 8115	228	100	0	0	147	70	0.7	3.4	25.9
21	*Rhodotorula pacifica*	JCM 10908	233	100	0	0	99	40.4	5.1	2	52.5
22	*Rhodotorula paludigena*	JCM 10292	303	100	0	0	158	63.3	3.2	4.4	29.1
23	*Rhodotorula paludigena*	JCM 10293	333	100	0	0	101	67.3	5	9.9	17.8
24	*Rhodotorula sphaerocarpa*	JCM 8202	236	100	0	0	103	61.1	3.9	4.9	30.1
25	*Rhodotorula sphaerocarpa*	JCM 3791	370	100	0	0	140	47.9	10.7	5	36.4
26	*Rhodotorula sphaerocarpa*	JCM 9055	228	100	0	0	121	75.3	7.4	7.4	9.9
27	*Rhodotorula taiwanesis*	JCM 3773	185	100	0	0	139	57.6	10.8	4.3	27.3
28	*Rhodotorula toruloides*	JCM 10021	219	100	0	0	173	70.5	6.4	2.3	20.8
29	*Rhodotorula toruloides*	JCM 10020	283	100	0	0	157	64.9	15.3	3.2	16.6
30	*Rhodotorula toruloides*	JCM 10022	199	100	0	0	164	85.4	4.3	1.2	9.1
31	*Rhodotorula toruloides*	JCM 10049	217	100	0	0	114	61.4	7.9	5.3	25.4
32	*Rhodotorula toruloides*	JCM 10297	165	100	0	0	101	70.3	5.9	4	19.8
33	*Rhodotorula toruloides*	JCM 10295	183	100	0	0	114	60.5	7.9	3.5	28.1
34	*Rhodotorula toruloides*	JCM 10298	89	100	0	0	77	81.8	3.9	1.3	13
35	*Rhodotorula toruloides*	JCM 24501	436	100	0	0	231	67.1	3.9	2.6	26.4
36	*Rhodotorula toruloides*	NBRC 10075	183	100	0	0	115	64.3	8.7	6.1	20.9
37	*Rhodotorula toruloides*	NBRC 10076	175	100	0	0	123	69.9	10.6	4.1	15.4
38	*Rhodotorula toruloides*	NBRP 10512	174	100	0	0	114	64	8.8	3.5	23.7
39	*Rhodotorula toruloides*	NBRP 10513	233	100	0	0	105	61.9	7.6	7.6	22.9
40	*Sporobolomyces salmonicolor*	JCM 1841	148	100	0	0	76	55.3	3.9	3.9	36.9
41	*Sporobolomyces salmonicolor*	JCM 8246	117	100	0	0	68	73.5	7.4	1.5	17.6
42	*Sporobolomyces salmonicolor*	JCM 21990	137	100	0	0	110	69.1	2.7	2.7	25.5
43	*Sporobolomyces blumeae*	JCM 10212	257	100	0	0	124	54.8	4.8	6.5	33.9
44	*Sporobolomyces carnicolor*	JCM 3766	332	100	0	0	134	53.7	3.7	15.7	26.9
45	*Sporobolomyces johnsonii*	JCM 1840	202	100	0	0	112	54.5	15.2	8.9	21.4
46	*Sporobolomyces koalae*	JCM 15063	194	100	0	0	140	58.6	5.7	7.1	28.6
47	*Sporobolomyces patagonicus*	JCM 16287	150	100	0	0	101	63.3	3	1	32.7
48	*Sporobolomyces phaffii*	JCM 11491	192	100	0	0	111	46	8.1	7.2	38.7
49	*Sporobolomyces roseus*	JCM 5353	347	100	0	0	146	35	3.4	4.1	57.5
50	*Sporobolomyces ruberrimus*	JCM 16303	128	100	0	0	109	54.2	6.4	6.4	33
51	*Sporobolomyces shibatanus*	JCM 3765	374	100	0	0	189	49.8	7.9	11.1	31.2

Fig. 3A

33 Trichosporonales yeasts used in the study are shown. Nuclear division phenotypes are observed in 20 species containing yeast from. The 20 species are colored by gray.

Trichosporonales

				All cells				Budded cells			
No.	Species	Strains	Total cell number	Yeast form (%)	Hyphal form (%)	Fission form (%)	Budded cell number	A nucleus in mother cell only (%)	A nucleus in daughter cell only (%)	A nucleus at bud neck (%)	Nuclei in both cells (%)
1	*Apiotrichum brassicae*	JCM 1599	154	0	100	0	0	0	0	0	0
2	*Apiotrichum domesticum*	JCM 9580	316	96.5	3.5	0	108	79.6	0	2.8	17.6
3	*Apiotrichum gamsii*	JCM 9941	69	0	100	0	0	0	0	0	0
4	*Apiotrichum gracile*	JCM 10018	215	0	4.2	95.8	0	0	0	0	0
5	*Apiotrichum laibachii*	JCM 2947	81	0	100	0	0	0	0	0	0
6	*Apiotrichum montevideense*	JCM 9937	174	0	100	0	0	0	0	0	0
7	*Apiotrichum porosum*	JCM 1458	53	3.8	96.2	0	2	0	0	0	0
8	*Apiotrichum veenhuisii*	JCM 10691	343	0	3.2	96.8	0	0	0	0	0

9	*Cutaneotrichosporon arboriforme*	JCM 14201	283	100	0	0	168	76.2	0	3	20.8
10	*Cutaneotrichosporon cavernicola*	HIS 02	258	100	0	0	167	76.5	2.94	11.2	9.36
11	*Cutaneotrichosporon cavernicola*	HIS 19	192	100	0	0	120	56.5	19.6	19.6	4.3
12	*Cutaneotrichosporon cavernicola*	HIS 631	240	100	0	0	133	81.9	4.5	6.8	6.8
13	*Cutaneotrichosporon cavernicola*	HIS 641	184	100	0	0	126	63.5	1.6	7.9	27
14	*Cutaneotrichosporon cavernicola*	HIS 712	187	100	0	0	105	49.5	20.2	18.2	12.1
15	*Cutaneotrichosporon aff. cavernicola*	HIS 471	173	100	0	0	110	70	5.5	11.8	12.7
16	*Cutaneotrichosporon curvatum*	JCM 1532	397	100	0	0	253	77.5	3.2	5.5	13.8
17	*Cutaneotrichosporon cutaneum*	JCM 1462	167	7.2	92.8	0	12	0	0	0	0
18	*Cutaneotrichosporon cyanovorans*	JCM 31833	321	100	0	0	211	72.1	0.9	5.7	21.3
19	*Cutaneotrichosporon daszewskae*	JCM 11166	232	100	0	0	165	80.6	0.6	8.5	10.3
20	*Cutaneotrichosporon dermatis*	JCM 11170	222	100	4.1	0	152	42.7	0.7	28.3	28.3
21	*Cutaneotrichosporon mucoides*	JCM 9939	356	100	0	0	267	79.4	1.5	4.5	14.6
22	*Cutaneotrichosporon spelunceum*	HIS 16	144	0	88.9	11.1	16	0	0	0	0
23	*Pascua guehoae*	JCM 10690	84	3.6	96.4	0	3	0	0	0	0
24	*Prillingera fragicola*	JCM 1530	253	100	0	0	171	74.9	5.8	5.8	13.5
25	*Trichosporon asahii*	JCM 2466	335	93.4	6.6	0	174	68.4	0.5	14.4	16.7
26	*Trichosporon asteroides*	JCM 2937	101	0	100	0	0	0	0	0	0
27	*Trichosporon coremiiforme*	JCM 2938	94	0	100	0	0	0	0	0	0
28	*Trichosporon faecale*	JCM 2941	160	87.5	12.5	0	65	46.2	7.6	0	46.2
29	*Trichosporon inkin*	JCM 9195	338	100	0	0	139	73.4	0.7	0	25.9
30	*Trichosporon ovoides*	JCM 9940	120	1.7	98.3	0	2	0	0	0	0
31	*Takashimella koratensis*	JCM 12878	454	100	0	0	120	71.6	4.2	4.2	20
32	*Takashimella tepidaria*	JCM 11965	426	100	0	0	253	75.5	8.7	3.6	12.2
33	*Vanrija humicola*	JCM 1457	259	100	0	0	89	67.5	0	11.2	21.3

Fig. 5A

Mother cell length is the distance from the edge of mother cell to the bud neck. Daughter cell length is the distance from the edge of daughter cell to the bud neck. Mother or daughter nucleus to the bud neck is the distance from the center of mother or daughter nucleus to the bud neck.

Time	Mother cell length (μm)	Daughter cell length (μm)	Mother nucleus to bud neck (μm)	Daughter nucleus to bud neck (μm)
0 min	8.31	1.81	3.37	
5 min	8.31	1.81	3.63	
10 min	8.31	2.07	3.89	
15 min	8.31	2.07	3.63	
20 min	8.31	2.07	3.37	
25 min	8.31	2.07	3.63	
30 min	8.57	2.07	3.89	
35 min	8.57	2.33	3.37	
40 min	8.31	2.59	3.89	
45 min	8.57	2.33	3.63	
50 min	8.57	2.59	3.89	
55 min	8.31	2.85	3.37	
60 min	8.31	3.11	3.37	
65 min	8.31	3.37	3.11	
70 min	8.31	3.89	3.11	
75 min	8.31	3.89	3.37	
80 min	8.31	4.15	3.11	
85 min	8.57	4.15	3.37	
90 min	8.31	4.93	2.85	
95 min	8.31	5.19	3.11	
100 min	8.31	5.19	3.11	
105 min	8.31	5.71	2.33	
110 min	8.57	5.71	2.59	
115 min	8.31	5.97	2.59	
120 min	8.31	6.23	2.07	
125 min	8.31	6.75	2.33	
130 min	8.31	7.27	2.07	
135 min	8.31	7.27	2.59	
140 min	8.31	7.53	2.07	
145 min	8.31	7.79	2.07	
150 min	8.31	8.31	1.03	
155 min	8.31	8.31	0.77	
160 min	8.31	8.57	0.77	
165 min	8.31	9.09	0	
170 min	8.31	9.61	4.67	3.89
175 min	8.31	9.61	4.93	3.37
180 min	8.31	9.61	5.19	4.41
185 min	8.31	9.87	4.93	4.67
190 min	8.31	9.87	4.93	4.41
195 min	8.57	9.87	4.93	4.41
200 min	8.57	10.12	5.19	4.41
205 min	8.31	10.64	4.93	4.15
210 min	8.05	11.42	4.93	4.41
215 min	8.05	11.94	4.93	4.41
220 min	8.05	12.2	4.93	4.41

Supplemental Table 2

Taxa and sequence accession numbers used in the study

Species	ITS (5.8S)	D1D2	SSU
Agaricomycotina			
Apiotrichum brassicae	AF444436	AF075521	AB001731
Apiotrichum domesticum	AF444414	AF075512	AB001754
Apiotrichum gamsii	KF036602	AF444708	KF036716
Apiotrichum gracile	AF444440	AF105399	AB001756
Apiotrichum laibachii	AF444421	AF075514	AB001760
Apiotrichum montevideense	AF444422	AF105397	AB001762
Apiotrichum porosum	AF414694	AF189833	AB051045
Apiotrichum veenhuisii	AF414693	AF105400	KF036724
Cutaneotrichosporon arboriforme	AB260936	AB260936	KF036619
Cutaneotrichosporon cavernicola	AB180195	AB180195	-
Cutaneotrichosporon curvatum	AF410467	AF189834	AB032626
Cutaneotrichosporon cutaneum	AF444325	AF075483	KF036712
Cutaneotrichosporon cyanovorans	JF680900	JF680899	-
Cutaneotrichosporon daszewskae	AB035580	AB126588	AB035582
Cutaneotrichosporon dermatis	AY143557	AY143555	AB035585
Cutaneotrichosporon mucoides	AF444423	AF075515	AB001763
Cutaneotrichosporon spelunceum	LN866279	LN866279	-
Cystofilobasidium capitatum	AF139627	AF075465	D12801
Filobasidium floriforme	AF190007	AF075498	D13460
Genolevuria amylolytica	KF036585	AY562134	KF036616
Holtermannia corniformis	AF410472	AF189843	AF053718
Mrakia frigida	AF144483	AF075463	D12802
Naganishia albida	AF145321	AF075474	AB032616
Papiliotrema aurea	AB035045	AB035041	AB085795
Papiliotrema laurentii	AF410468	AF075469	AB032640
Pascua guehoae	AF410476	AF105401	KF036717
Phaffia rhodozyma	AF139629	AF189871	KF036689
Prillingera fragicola	AB035588	AB126585	AB035588
Solicoccozyma aeria	AF145324	AF075486	AB032614
Takashimella koratensis	AY919655	AY313006	AY863105
Takashimella tepidaria	AB094045	AB094046	-
Tausonia pullulans	AF444417	EF551318	AB001766
Tremella fuciformis	AF444316	AF075476	KF036701
Trichosporon asahii	AY055381	AF105393	AB001726
Trichosporon asteroids	AF444416	AF075513	AB001729
Trichosporon coremiiforme	AF444434	AF139983	AB001727

Trichosporon faecale	AF444419	AF105395	AB001728
Trichosporon inkin	AF444420	AF105396	AB001757
Trichosporon ovoides	AF444439	AF075523	AB001765
Vanrija humicola	AF410470	AF189836	AB032637
Pucciniomycotina			
Ballistosporomyces taupoensis	AF444592	AF177413	D66886
Bannoa hahajimensis	AB035897	AB082571	AB035897
Bensingtonia ciliata	AF444563	AF189887	D38233
Buckleyzyma aurantiaca	AF444538	AF189921	KJ708436
Chionosphaera cuniculicola	KJ778640	KJ708465	KJ708368
Curvibasidium cygneicollum	AF444490	AF189928	KJ708423
Cystobasidiopsis lophatheri	AB126046	AB124561	AB126046
Cystobasidium minutum	AF190011	AF189945	D45367
Erythrobasidium hasegawianum	AF444522	AF189899	D12803
Glaciozyma antarctica	AF444529	AF189906	DQ785788
Kondoa malvinella	AF444498	AF189903	D13776
Kurtzmanomyces insolitus	AF444594	AF177408	KJ708424
Leucosporidium scottii	AF444495	AF070419	X53499
Microsporomyces magnisporus	AB112078	AB111954	KJ708428
Mixia osmundae	DQ831010	DQ831009	D14163
Mycogloea nipponica	KJ778629	KJ708456	KJ708370
Naohidea sebacea	DQ911616	DQ831020	KP216515
Phyllozyma subbrunnea	AF444549	AF189997	AB021691
Pseudobensingtonia ingoldii	AF444519	AF189888	D38234
Puccinia graminis f. sp. *tritici*	AF468044	AF522177	AY125409
Rhodosporidiobolus azoricus	AB073229	AF321977	AB073269
Rhodosporidiobolus fluvialis	AY015432	AF189915	AB073272
Rhodosporidiobolus lusitaniae	AY015430	AF070423	AB073274
Rhodosporidiobolus microsporus	AF444535	AF070436	KJ708441
Rhodosporidiobolus nylandii	AB030323	AF387123	AB030319
Rhodosporidiobolus odoratus	KJ778638	AF387125	KJ708427
Rhodosporidiobolus poonsookiae	AB030327	AF387124	AB030320
Rhodosporidiobolus ruineniae	AF444491	AF070434	AB021693
Rhodotorula alborubescens	AB030342	AF207886	KJ708440
Rhodotorula araucariae	AF444510	AF070427	KJ708435
Rhodotorula babjevae	AF444542	AF070420	AB073270
Rhodotorula dairenensis	AF444501	AY033552	KJ708411
Rhodotorula diobovata	AF444502	AF070421	AB073271
Rhodotorula glutinis	AF444539	AF070429	X69853
Rhodotorula graminis	AF444505	AF070431	X83827

Rhodotorula kratochvilovae	AF444520	AF071436	AB073273
Rhodotorula mucilaginosa	AF444541	AF070432	AB021668
Rhodotorula pacifica	AB026006	AB026006	KJ708397
Rhodotorula paludigena	AF444492	AF070424	KJ708422
Rhodotorula sphaerocarpa	AF444499	AF070425	AB073275
Rhodotorula taiwanesis	GU646862	GU646863	KJ708409
Rhodotorula toruloides	AF444489	AF070426	X60180
Ruinenia rubra	AF444550	AF189992	AB021686
Sakaguchia dacryoidea	AF444597	AF189972	D13459
Sporobolomyces blumeae	AB030331	AY213010	AB030321
Sporobolomyces carnicolor	AY069991	AY070008	KJ708434
Sporobolomyces johnsonii	AY015431	AF070435	L22261
Sporobolomyces koalae	EU276008	EU276011	KP216519
Sporobolomyces patagonicus	AY552328	AY158655	KJ708421
Sporobolomyces phaffii	AY069995	AY070011	KJ708404
Sporobolomyces roseus	AY015438	AF070441	X60181
Sporobolomyces ruberrimus	AY015439	AF070442	KJ708402
Sporobolomyces salmonicolor	AY015434	AF070439	AB021697
Sporobolomyces shibatanus	AF417115	AF070437	AB021694
Sterigmatomyces elviae	AF444551	AF177415	KJ708432
Symmetrospora marina	AF444504	AF189944	AB126645
Ustilaginomycotina			
Gjaerumia minor	AJ235287	KP322989	KP322972
Robbauera albescens	AJ235289	KP322986	KP322968
